# Unraveling the pathophysiology of schizophrenia: insights from structural magnetic resonance imaging studies

**DOI:** 10.3389/fpsyt.2023.1188603

**Published:** 2023-05-19

**Authors:** Mohammed Jajere Adamu, Li Qiang, Charles Okanda Nyatega, Ayesha Younis, Halima Bello Kawuwa, Adamu Halilu Jabire, Sani Saminu

**Affiliations:** ^1^Department of Electronic Science and Technology, School of Microelectronics, Tianjin University, Tianjin, China; ^2^Department of Computer Science, Yobe State University, Damaturu, Nigeria; ^3^Department of Information and Communication Engineering, School of Electrical and Information Engineering, Tianjin University, Tianjin, China; ^4^Department of Electronics and Telecommunication Engineering, Mbeya University of Science and Technology, Mbeya, Tanzania; ^5^Department of Biomedical Engineering and Scientific Instruments, School of Precision Instruments and Optoelectronics Engineering, Tianjin University, Tianjin, China; ^6^Department of Electrical and Electronics Engineering, Taraba State University, Jalingo, Nigeria; ^7^Department of Biomedical Engineering, University of Ilorin, Ilorin, Nigeria

**Keywords:** schizophrenia, structural MRI, voxel-based morphometry, gray matter, white matter, cerebrospinal fluid, statistical parametric mapping

## Abstract

**Background:**

Schizophrenia affects about 1% of the global population. In addition to the complex etiology, linking this illness to genetic, environmental, and neurobiological factors, the dynamic experiences associated with this disease, such as experiences of delusions, hallucinations, disorganized thinking, and abnormal behaviors, limit neurological consensuses regarding mechanisms underlying this disease.

**Methods:**

In this study, we recruited 72 patients with schizophrenia and 74 healthy individuals matched by age and sex to investigate the structural brain changes that may serve as prognostic biomarkers, indicating evidence of neural dysfunction underlying schizophrenia and subsequent cognitive and behavioral deficits. We used voxel-based morphometry (VBM) to determine these changes in the three tissue structures: the gray matter (GM), white matter (WM), and cerebrospinal fluid (CSF). For both image processing and statistical analysis, we used statistical parametric mapping (SPM).

**Results:**

Our results show that patients with schizophrenia exhibited a significant volume reduction in both GM and WM. In particular, GM volume reductions were more evident in the frontal, temporal, limbic, and parietal lobe, similarly the WM volume reductions were predominantly in the frontal, temporal, and limbic lobe. In addition, patients with schizophrenia demonstrated a significant increase in the CSF volume in the left third and lateral ventricle regions.

**Conclusion:**

This VBM study supports existing research showing that schizophrenia is associated with alterations in brain structure, including gray and white matter, and cerebrospinal fluid volume. These findings provide insights into the neurobiology of schizophrenia and may inform the development of more effective diagnostic and therapeutic approaches.

## 1. Introduction

Schizophrenia (SZ) is a debilitating psychiatric disorder that affects a large population worldwide (1), with a higher prevalence in males and a peak onset in the late teenage years or early adulthood. The disorder has a significant impact on the individual’s ability to function and can lead to disability and increased mortality rates (2).

The exact cause of schizophrenia remains unknown, although research has demonstrated that a combination of genetic, environmental, and neurological variables has a role in the development of schizophrenia, the origins of the disorder are complicated and not entirely understood (3, 4). Schizophrenia is known to have a strong genetic component, with heritability estimated to be around 80%. Several genetic loci related with schizophrenia have been discovered recently through genome-wide association studies (GWAS), including genes implicated in synapse function, immune system function, and calcium signaling (5, 6). A recent study has also suggested that the effects of genetic risk factors for schizophrenia may be modified by environmental factors (7). Numerous environmental factors have been implicated in the development of schizophrenia. Prenatal and perinatal factors such as maternal infection, stress, and malnutrition have been linked to an increased risk of schizophrenia (8, 9). Childhood trauma, including physical and sexual abuse, has also been associated with an increased risk of schizophrenia (10). Other environmental factors such as cannabis use, urbanization, and migration have also been shown to increase the risk of developing schizophrenia (11–13). Schizophrenia is characterized by numerous neurobiological abnormalities, including changes in neurotransmitter signaling, aberrant brain development, and altered synaptic connectivity. Dysregulation of dopamine neurotransmission is a well-established feature of schizophrenia, with recent evidence also implicating alterations in glutamate and GABA neurotransmitter systems (13, 14). Abnormalities in immune function and oxidative stress have also been implicated in the pathophysiology of schizophrenia (15).

Schizophrenia is characterized by positive, negative, and cognitive symptoms. Positive symptoms include experiences that are added to a person’s normal range of experiences, such as hallucinations, delusions, and disorganized speech. Negative symptoms refer to experiences that are taken away from a person’s normal range of experiences, such as apathy, social withdrawal, and lack of motivation or pleasure in activities. Cognitive symptoms refer to problems with attention, memory, and decision-making (16, 17). These symptoms can vary in severity and can have a significant impact on the individual’s daily functioning.

The treatment of schizophrenia typically involves a combination of medication, therapy, and social support. Antipsychotic medications are frequently used in conjunction with therapy to treat positive symptoms in schizophrenia, while psychosocial interventions can help improve the negative and cognitive symptoms (18). Cognitive behavioral therapy and social skills training are mostly used to treat negative symptoms (19, 20). Antipsychotic medications are the cornerstone of pharmacological treatment for schizophrenia. The first-generation antipsychotics (FGAs) were introduced in the 1950s and 1960s, followed by second-generation antipsychotics (SGAs) in the 1990s. While both FGAs and SGAs are effective in reducing positive symptoms of schizophrenia, SGAs have fewer side effects and are better tolerated by patients (21). Recently, several new antipsychotic medications, such as lumateperone and cariprazine, have been approved for the treatment of schizophrenia (22). People with schizophrenia have shown to benefit from psychosocial interventions including cognitive behavioral therapy (CBT) and social skills training (SST) in terms of lowering negative symptoms and enhancing social functioning (23). Family interventions, which aim to improve family communication and reduce stress, have also been shown to be effective in reducing relapse rates and improving social functioning in patients with schizophrenia (24). Integrated treatment, which combines pharmacological and psychosocial interventions, has been shown to be particularly effective in improving outcomes for individuals with schizophrenia (25). Despite the availability of effective treatments, many individuals with schizophrenia do not receive adequate care due to a lack of access to mental health services or the stigma associated with the disorder. Additionally, the heterogeneity of the disorder can make it difficult to identify the most effective treatment approach for each individual (26).

Individuals with schizophrenia are at increased risk for developing comorbid medical conditions such as hypertension and dyslipidemia, which can further complicate the management of their condition. The increased risk for other mental illnesses such as depression and anxiety are also a significant challenge in the treatment of schizophrenia (27). Dissociative symptoms, such as depersonalization and derealization, are common in individuals with schizophrenia and can complicate the diagnosis and treatment of the disorder (28). The historical changes in the diagnosis of schizophrenia, which has been reclassified multiple times, have also contributed to the challenges in understanding and treating the disorder (29). Schizophrenia can be a debilitating disorder, although many people with the illness can have fulfilling lives with the right care and support (30).

Neural substrate of schizophrenia involves various brain regions, including the prefrontal cortex (PFC), temporal lobe, and limbic system. However, frontal lobe dysfunction is one of the most consistent findings in schizophrenia. Studies have demonstrated decreased activation and structural abnormalities in the PFC of individuals with schizophrenia, which is associated with cognitive impairment and negative symptoms (31). Additionally, abnormalities in the connectivity between the PFC and other brain regions have also been observed in schizophrenia. These findings suggest that the PFC plays a crucial role in the pathophysiology of schizophrenia, and further investigation of this brain region may lead to a better understanding of the disorder and the development of more effective treatments (4).

One area of investigation in schizophrenia research has been structural brain abnormalities, which have been found to be present in many patients with the disorder (32). Specifically, abnormalities have been found in brain regions that are involved in a range of cognitive and emotional functions, including attention, memory, language, and social cognition, which are frequently impaired in individuals with schizophrenia (33). Structural brain abnormalities in brain disorder are reflected in changes in the brain’s gray matter, white matter, and cerebrospinal fluid; GM, WM, and CSF (34). The GM abnormalities in schizophrenia are particularly noteworthy, as they are thought to reflect neuronal loss, decreased dendritic arborization, and synaptic dysfunction (35). In contrast, WM abnormalities in schizophrenia may reflect altered myelination, reduced axonal density, and disrupted connectivity between brain regions (35). CSF abnormalities, such as ventricular enlargement and CSF kynurenine (KYN) level, have been linked to brain atrophy and cognitive decline in multiple brain disorder including schizophrenia (14, 36–38).

A wide range of neuroimaging techniques have been developed to detect abnormalities and provide insights into the neural underpinnings of schizophrenia (SZ). Gray matter (GM) abnormalities in SZ can be detected using structural magnetic resonance imaging (sMRI), which measures brain tissue volume and density. Studies using sMRI have reported reduced GM volume in various brain regions, including the prefrontal cortex and hippocampus, in SZ patients compared to healthy controls (39). White matter (WM) abnormalities in SZ can be detected using diffusion tensor imaging (DTI), which measures WM microstructure and connectivity. Studies using DTI have reported decreased WM integrity in various brain regions, including the corpus callosum, uncinate fasciculus, and superior longitudinal fasciculus, in SZ patients compared to healthy controls (40). Tractography, a technique that visualizes and measures WM tracts in the brain, has revealed disrupted WM connectivity in SZ, with significant differences observed in certain regions of the brain (41, 42). Cerebrospinal fluid (CSF) abnormalities in SZ can be detected using sMRI, which measures CSF volume. Studies using sMRI have reported increased CSF volume in various brain regions, in SZ patients compared to healthy controls (43).

One commonly used MRI analysis method is voxel-based morphometry (VBM), which is a powerful and flexible technique for quantifying differences in GM, WM, and CSF between groups of individuals (44). Compared to other structural brain imaging analysis techniques, VBM allows for voxel-by-voxel analysis of the entire brain, making it possible to identify widespread structural abnormalities that may not be apparent using other techniques (44). It is highly sensitive and can detect subtle changes in brain structure that may be missed by other methods (44). Several recent studies have reported gray matter and white matter abnormalities in patients with schizophrenia. A study by (45) found that patients with schizophrenia had decreased gray matter volume in several brain regions, including the anterior cingulate cortex (ACC), gyrus rectus, and insula. The study also found decreased white matter integrity in the inferior temporal gyrus and superior temporal gyrus (STG), as well as the superior frontal gyrus. However, no CSF volume difference was detected. The findings reveal that the white and gray matter abnormalities were associated with the psychopathology of the patients with schizophrenia. Another recent study by (46) found that patients with schizophrenia had significant decreases in gray matter volume in the anterior cingulate cortex, insula, and thalamus. The study also found that patients had difference in white matter integrity in several regions, including the corpus callosum, fornix, and the superior longitudinal fasciculus.

The rationale for this study is to further investigate the structural brain differences between individuals with schizophrenia and healthy controls using voxel-based morphometry (VBM) analysis. Despite the existence of previous VBM studies, it was noted that the results of these studies have not always been consistent, and some have been limited by small sample sizes or methodological differences. Therefore, this study aims to contribute to the field by using a relatively larger sample size and standardized methods to increase the reliability of the findings. The novelty of this study lies in its use of a larger sample size from the COBRE project (47) and its standardized VBM analysis methods. Additionally, the study’s inclusion of both gray matter (GM), white matter (WM), and cerebrospinal fluid (CSF) volumes in the analysis provides a more comprehensive understanding of the structural brain differences between individuals with schizophrenia and healthy controls.

In this investigation, we used structural MRI datasets from the International Neuroimaging Data-Sharing Initiative (INDI) project (47), to perform a voxel-based morphometry (VBM) analysis to examine the GM, WM, and CSF in the brains of 72 schizophrenia patients and 74 healthy controls. The study’s objective was to use the VBM technique to examine the differences in brain structure between people with schizophrenia and healthy controls. This study provides further evidence for the structural brain differences in individuals with schizophrenia compared to healthy controls, particularly in the frontal and temporal lobe. These findings have implications for the understanding and treatment of schizophrenia and may aid in the development of more targeted interventions. Additionally, the standardized methods used in this study may serve as a reference for future studies in the field.

## 2. Materials and methods

### 2.1. Participants

Participants were recruited from the Center for Biomedical Research Excellence (COBRE) database, which is part of the Mind Research Network for Neurodiagnostic Discovery (47). The study included 72 patients with schizophrenia and 74 healthy control participants who were matched for age, gender, and education level. All participants provided informed consent prior to the study and were compensated for their time.

Inclusion criteria for the schizophrenia group included a DSM-IV diagnosis of schizophrenia, as confirmed by the Structured Clinical Interview for DSM-IV (SCID), age between 18 and 65 years, and stable medication use for at least 4 weeks prior to enrollment. Exclusion criteria for both groups included a history of neurological disorders, intellectual disability, a traumatic head injury that caused them to lose consciousness for more than five minutes, or substance abuse within the past 12 months, and contraindications for MRI scanning (47). Tables 1, 2 provide a detailed primary diagnostic characterization of schizophrenic patients and healthy controls and demographic characteristics of participants.

**Table 1:** Primary diagnostic characterization of schizophrenic patients and healthy controls.

DSM Code	Number
Patients:	
Dementia of the Alzheimer ‘s type, with late onset, with delirium (290.3)	1
Catatonic type (295.2)	1
Disorganized type (295.1)	3
Bipolar type I (295.7)	1
Residual type (295.6)	12
Paranoid type (295.3)	41
Undifferentiated type (295.9)	5
Schizoaffective Disorder type (295.7)	5
Depresses type (295.7)	1
Unspecified type schizophrenia chronic state (295.92)	1
Bipolar Disorder type I, Most Recent Episode Mixed, In Full Remission (296.4)	1
Healthy Controls:	
Depressive Disorder type, Not Otherwise Specified (311)	1
Major Depressive Disorder, Single Episode, In Partial Remission (296.26)	1
Other Healthy Controls (none)	72

DSM, Diagnostic and statistical manual of mental disorders.

**Table 2:** Demographic Characteristics of Participants.

Parameter	SZ *n* = 72	HC *n* = 74	*p*-value
Age (years)	38.17 ± 13.89	35.82 ± 11.58	0.270^1^
Handedness			
Right/Left/Both	60/10/2	71/1/2	0.106^2^
Sex (Female/Male)	14/58	23/51	0.106^2^
IQ	(*n* = 68)	(*n* = 67)	
Verbal	97.88 ± 16.73	106.79 ± 11.16	<0.001^1^
Performance	102.68 ± 16.64	114.03 ± 12.32	<0.001^1^
Sum	99.59 ± 16.86	108.33 ± 11.83	<0.001^1^
Education (years)	12.99 ± 1.84	13.52 ± 1.75	0.089^1^
Illness duration (years, *n* = 71)	16.03 ± 12.41		
PANSS (*n* = 72)			
Positive scale	14.96 ± 4.83		
Negative scale	14.53 ± 4.83		
General	29.22 ± 8.34		

1 Two-sample *t*-test. Data are shown in mean ± SD.

2 Chi-square test.

### 2.2. MRI acquisition

All participants underwent MRI scanning at the Mind Research Network using a 3 T Siemens Magnetom Trio Tim scanner with a 12-channel head coil. High-resolution structural MRI images were acquired using a T1-weighted MPRAGE sequence (TR = 1900 ms, TE = 2.32 ms, flip angle = 9°, FOV = 256 mm, matrix size = 256 × 256, slice thickness = 1 mm). Resting-state fMRI data were acquired using a gradient-echo EPI sequence (TR = 2000 ms, TE = 27 ms, flip angle = 90°, FOV = 240 mm, matrix size = 64 × 64, slice thickness = 3.5 mm) during a 10-min scan. Participants were instructed to keep their eyes closed and remain still during the scan.

In addition, demographic and clinical data were collected from all participants, including age, gender, education level, and symptom severity (as measured by the Positive and Negative Syndrome Scale). All data were stored in a secure database and de-identified prior to analysis.

### 2.3. Voxel-based morphometry

#### 2.3.1. Image preprocessing

The preprocessing was performed using the SPM12 package’s integrated CAT12 toolbox (48, 49) and MATLAB (50). The DARTEL technique was used to normalize and segment the 3D T1-weighted NIFTI MRI images into tissue categories for the gray matter (GM), white matter (WM), and cerebrospinal fluid (CSF) using a default resolution of 1.5 mm and in MNI space. The generated maps were modulated with Jacobian determinant maps and smoothed with an 8-mm FWHM Gaussian kernel to maintain GM volume in native space. All operations, including segmentation, normalization, and modulation, were carried out automatically *via* the CAT12 toolkit. The native space volume estimations of the GM, WM, and CSF maps were computed using the total intracranial volume (TIV) as a covariate.

#### 2.3.2. Data analysis

**Figure 1:**
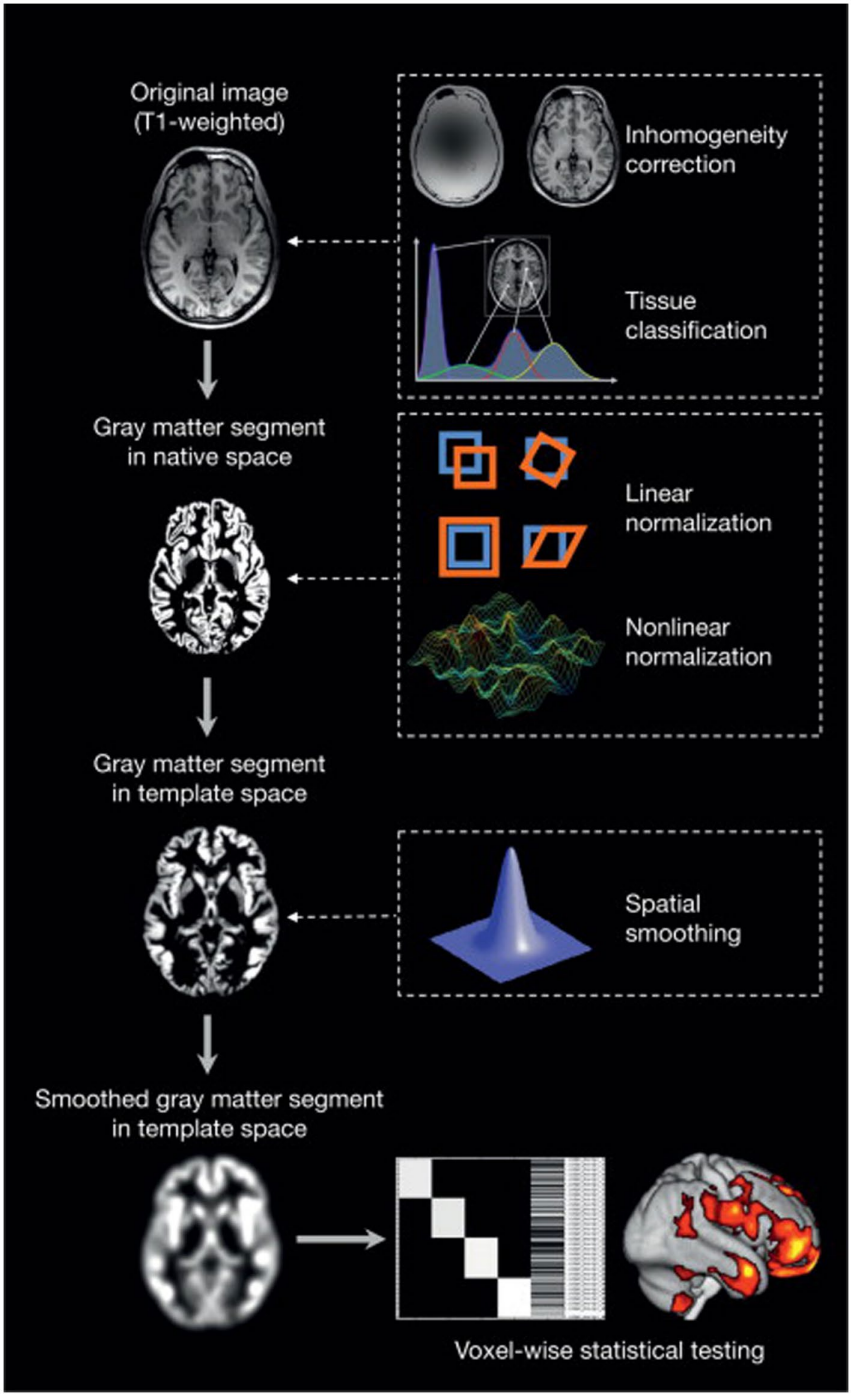
An overview of voxel-based morphometry analyses (52).

A two-tailed *t*-test was generated using family-wise error correction and a value of *p* of less than 0.05. The 100-voxel extent threshold was used. In MATLAB, the xjview (51) toolkit was used to visualize the brain regions with significant differences by assigning a pseudo-color and to display the activation volume (cluster) and activation intensity (statistically analyzed with a *t*-test and represented as *T* value, proportional to intensity). A graphic illustration of the VBM analysis procedure is shown in Figure 1.

## 3. Results

### 3.1. Demographic and neuropsychological characterization of participants

The subjects’ clinical information is presented in Table 2, the results of two-sample *t*-tests indicate that there was no significant difference between the age, sex, handedness, and education years of the two groups (*p* > 0.05). However, a significant difference was observed in IQ (*p* < 0.05) as determined by the two-sample *t*-tests. Table 2 contains the PANSS scale scores for the patient group and the primary diagnosis information for the schizophrenia (SZ) patient group and the healthy control (HC) group. Additional data can be found in the COBRE INDI Additional data in the supplementary materials (47).

### 3.2. Voxel-based morphometry analysis

**Figure 2:**
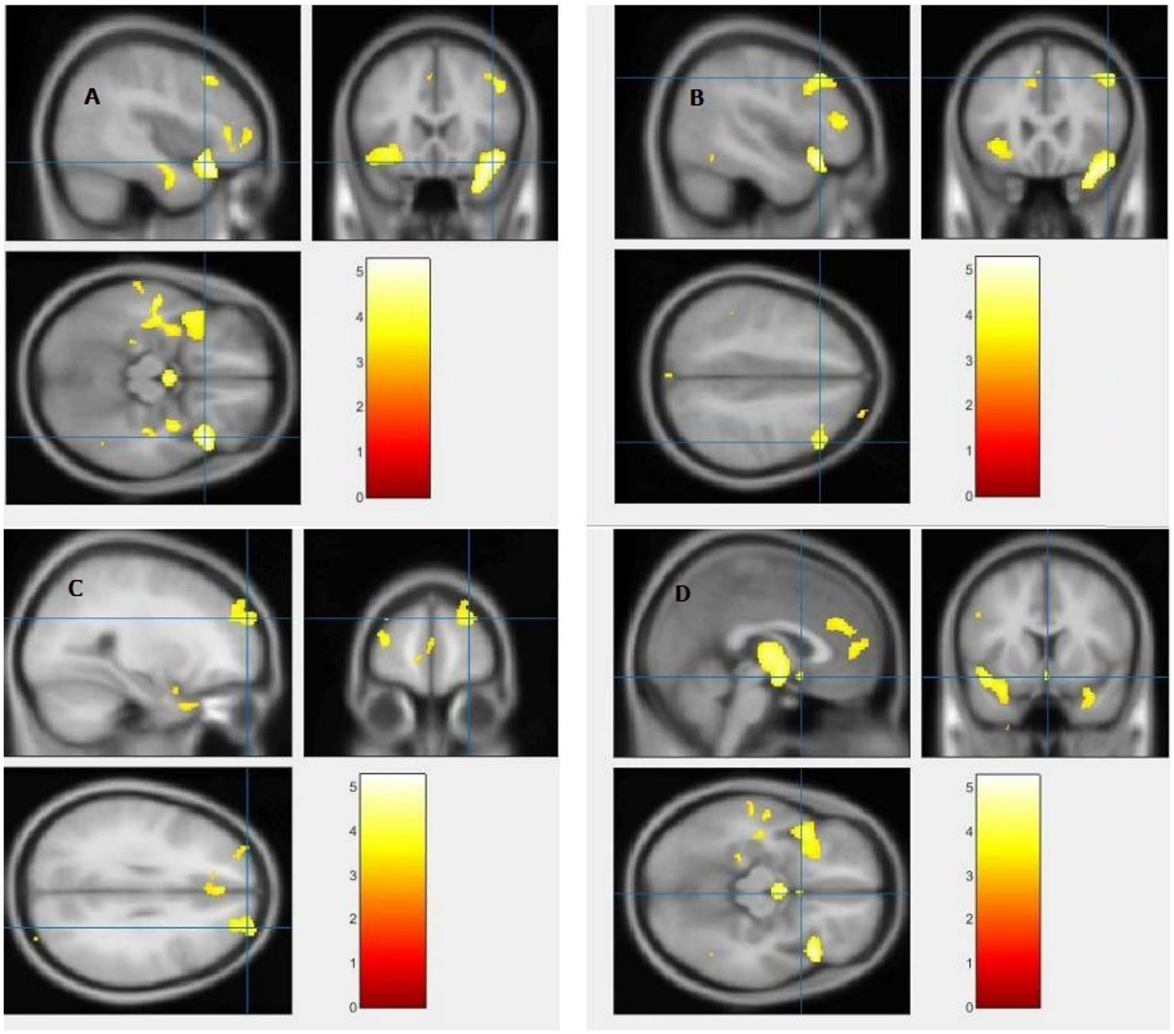
Voxel-based morphometric analyses identified gray matter (GM) alterations in the Right inferior frontal gyrus **(A)**, Right middle frontal gyrus **(B)**, Right superior frontal gyrus **(C)** and Right subcallosal gyrus **(D)**, with statistical significance at *p* < 0.05 and an extent threshold of *K* = 100 when SZ < HC.

**Figure 3:**
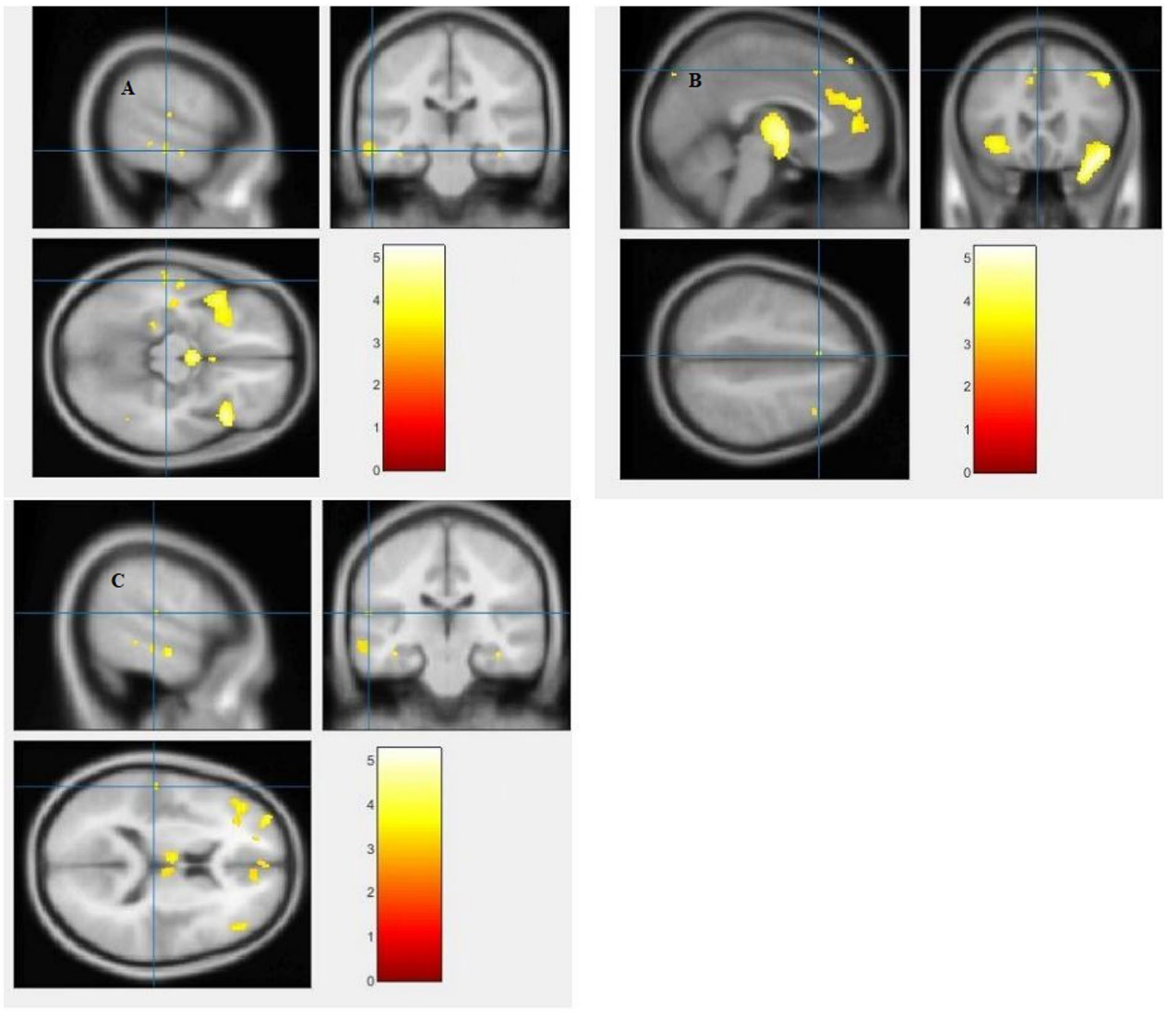
Voxel-based morphometric analyses identified gray matter (GM) alterations in the Left middle temporal gyrus **(A)**, Left cingulate gyrus **(B)**, and Left postcentral gyrus **(C)**, with statistical significance at *p* < 0.05 and an extent threshold of *K* = 100 when SZ < HC.

**Figure 4:**
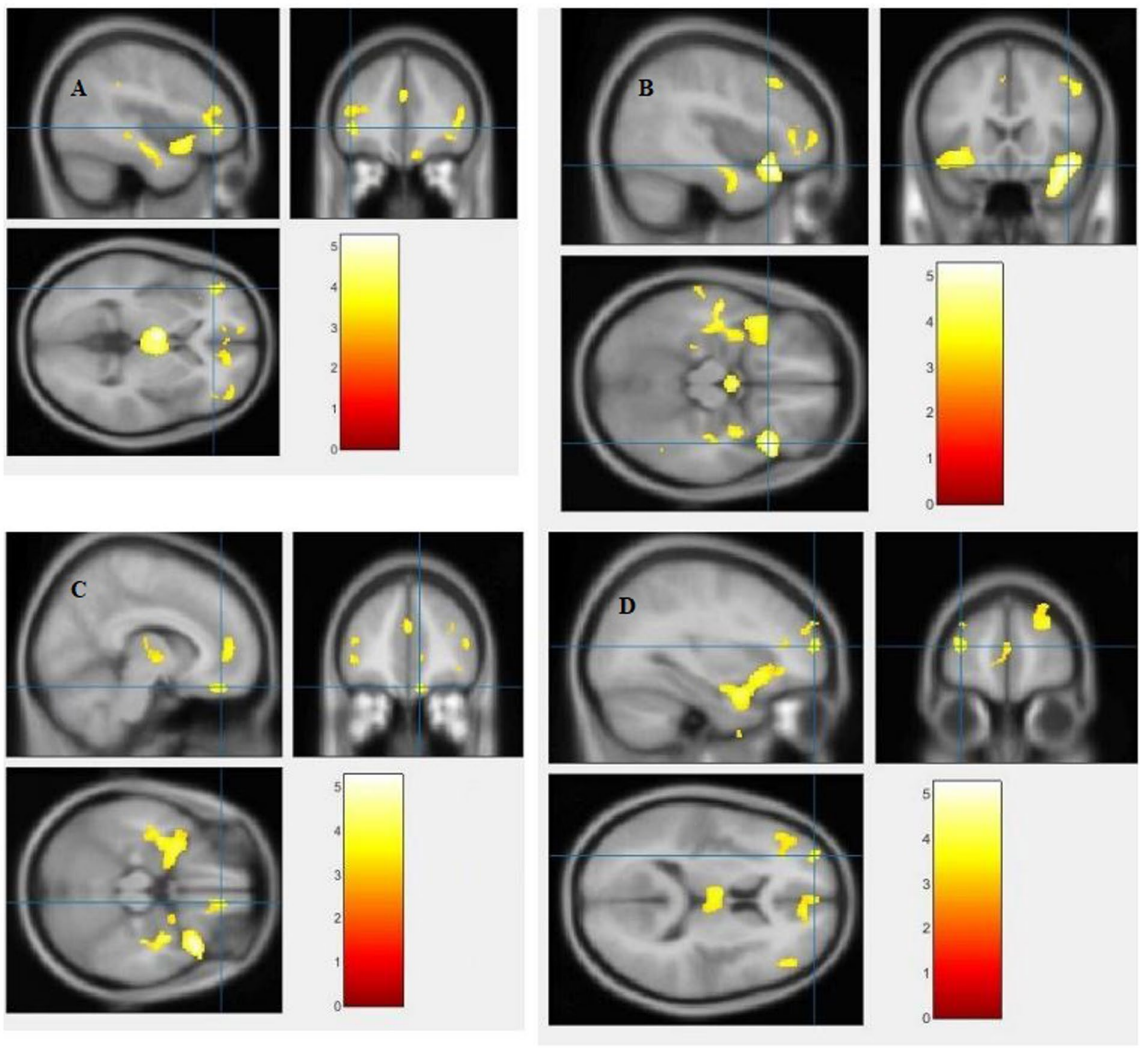
Voxel-based morphometric analyses identified white matter (WM) alterations in the Left inferior frontal gyrus **(A)**, Right inferior frontal gyrus **(B)**, Right sub-gyral **(C)** and Left middle frontal gyrus **(D)**, with statistical significance at *p* < 0.05 and an extent threshold of *K* = 100 when SZ < HC.

**Figure 5:**
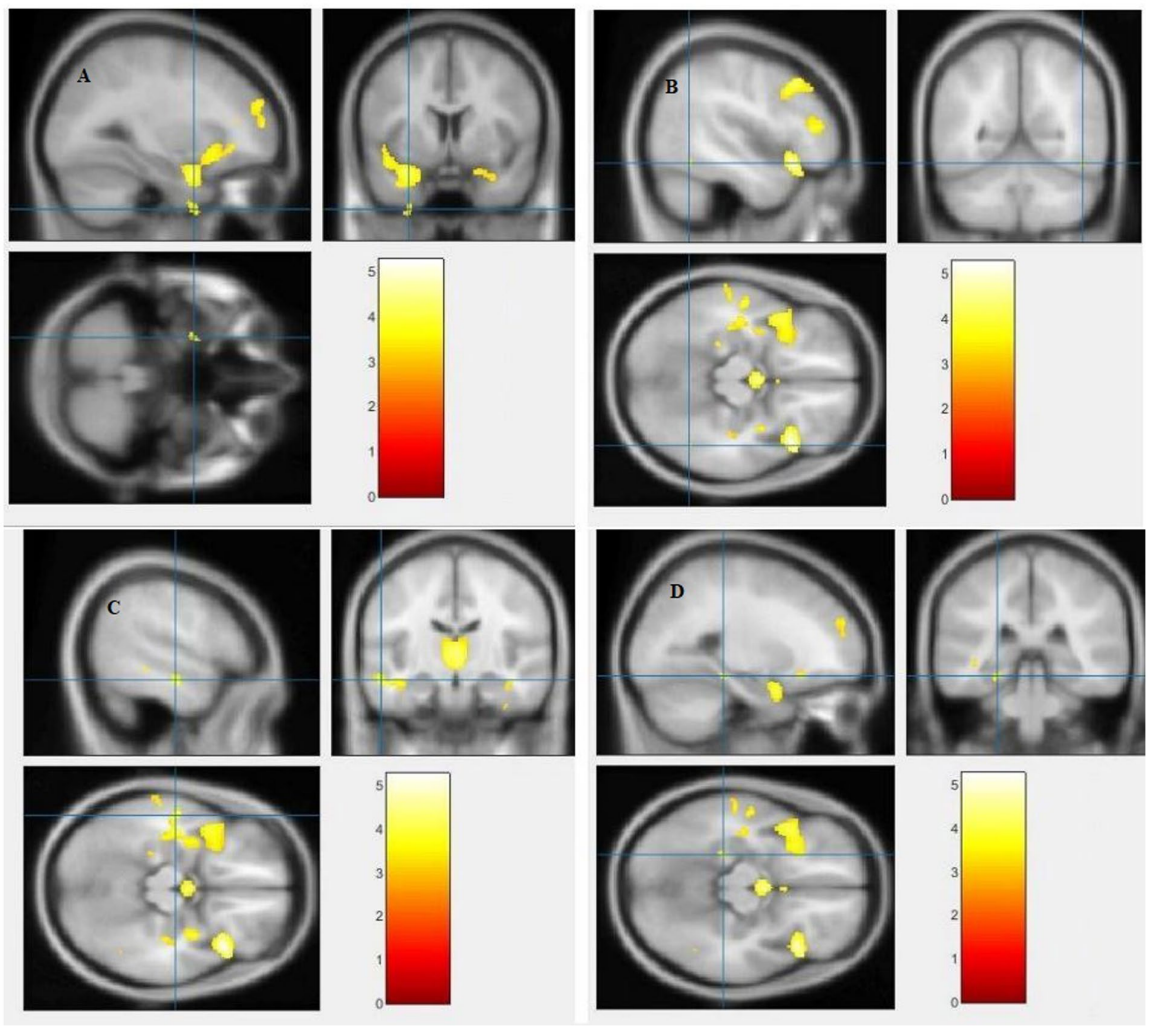
Voxel-based morphometric analyses identified white matter (WM) alterations in the Left superior temporal gyrus **(A)**, Right fusiform gyrus **(B)**, Left middle temporal gyrus **(C)** and Left parahippocampal gyrus **(D)**, with statistical significance at *p* < 0.05 and an extent threshold of *K* = 100 when SZ < HC.

**Figure 6:**
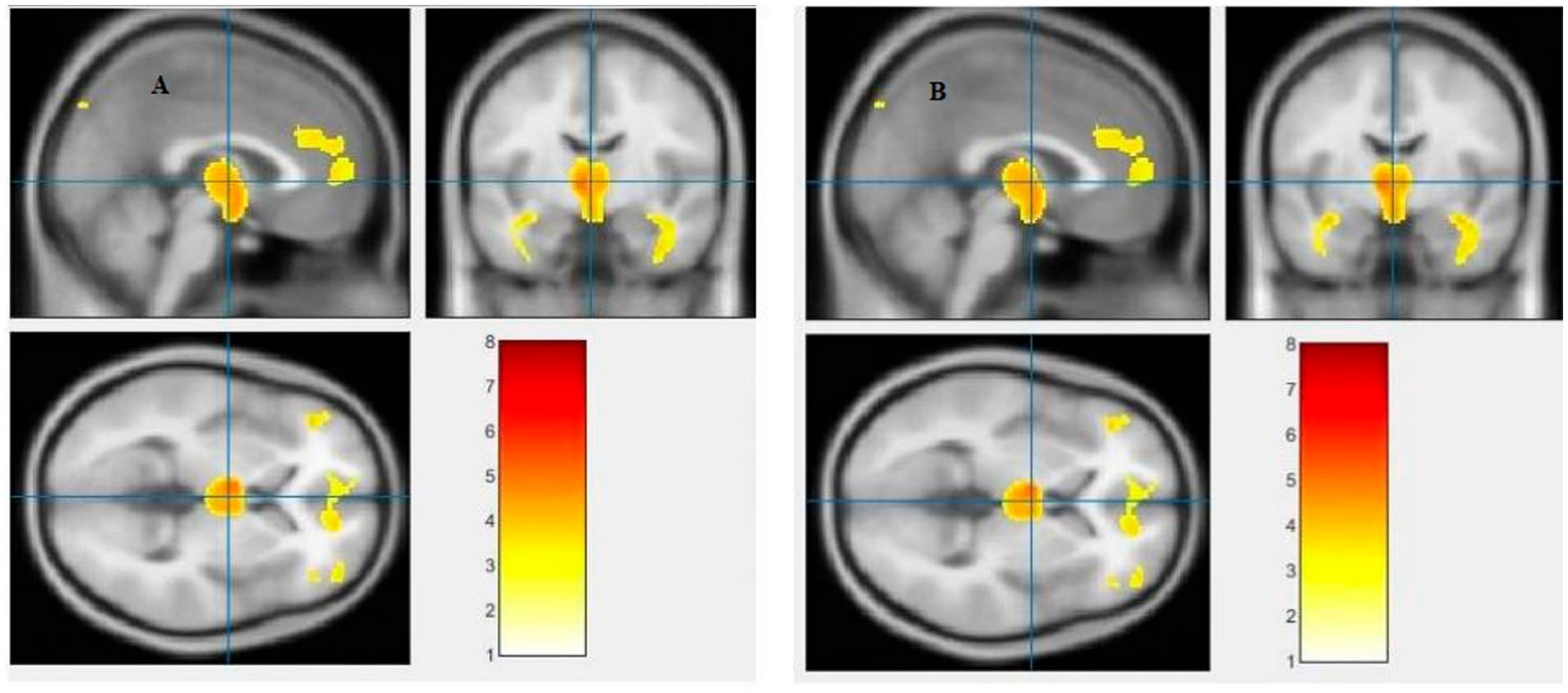
Voxel-based morphometric analyses identified cerebrospinal fluid (CSF) alterations in the Left third ventricle **(A)**, and Left lateral ventricle **(B)**, with statistical significance at *p* < 0.05 and an extent threshold of *K* = 100 when SZ > HC.

The study used a voxel-by-voxel analysis with family-wise error correction method to compare the gray matter, white matter, and cerebrospinal fluid volumes between schizophrenic patients and healthy controls. The significance level was set at *p* < 0.05 in a t test and extent threshold K = 100. The results showed significant reductions in gray matter volume in multiple brain regions in schizophrenic patients compared to healthy controls, including the right inferior frontal gyrus, right middle frontal gyrus, right superior frontal gyrus, right subcallosal gyrus, left middle temporal gyrus, left postcentral gyrus, and left cingulate gyrus (Table 3; Figures 2, 3). Moreover, significant reductions in white matter volume were observed in several brain regions in schizophrenic patients, including the left parahippocampal gyrus, left superior temporal gyrus, right fusiform gyrus, left middle temporal gyrus, left inferior frontal gyrus, right inferior frontal gyrus, right sub-gyral, and right middle frontal gyrus (Table 4; Figures 4, 5). White matter plays a critical role in connecting different brain regions and facilitating communication between them. Finally, the analysis revealed that schizophrenic patients had significant increases in cerebrospinal fluid volume in the left third ventricle and left lateral ventricle regions compared to healthy controls (Table 5; Figure 6). Figures 2-6 and Tables 3-5 contain detailed information on the brain regions and peak voxel coordinates.

**Table 3:** Evidence of gray matter alteration detected by voxel-based morphometry.

Contrast	Regions	L/R	Voxels Size	Clusters Number	MNI coordinates(mm)			Voxel level			
					*X*	*Y*	*Z*	*T*-value	*Z*-value	*p*_FWE corrected_	*p*_FDR corrected_
SZ < HC	Inferior Frontal Gyrus	R	2,601	2	42	20	−17	5.24	5.01	0.009	0.006
	Middle Frontal Gyrus	R	511	25	47	24	42	4.31	4.17	0.242	0.011
	Superior Frontal Gyrus	R	695	22	27	59	29	3.99	3.87	0.538	0.018
	Subcallosal Gyrus	R	29	9	2	11	−14	3.46	3.39	0.969	0.037
	Middle Temporal Gyrus	L	158	5	−59	−26	−14	3.45	3.37	0.973	0.038
	Postcentral Gyrus	L	9	18	−57	−24	14	3.35	3.28	0.990	0.042
	Cingulate Gyrus	L	16	28	−3	23	47	3.29	3.22	0.995	0.045
SZ > HC	–	–	–	–	–	–	–	–	–	–	–

**Table 4:** Evidence of white matter alteration detected by voxel-based morphometry.

Contrast	Regions	L/R	Voxels Size	Clusters Number	MNI coordinates(mm)			Voxel level			
					*X*	*Y*	*Z*	*T*-value	*Z*-value	*p*_FWE corrected_	*p*_FDR corrected_
SZ < HC	Middle Temporal Gyrus	L	2,957	3	−54	−15	−17	4.38	4.23	0.195	0.010
	Inferior Frontal Gyrus	L	576	14	−42	38	0	4.16	4.03	0.361	0.013
	Inferior Frontal Gyrus	R	380	11	45	36	9	3.86	3.76	0.677	0.022
	Sub-Gyral	R	169	4	9	42	−21	3.86	3.75	0.682	0.022
	Middle Frontal Gyrus	L	477	17	−32	57	12	3.87	3.73	0.706	0.023
	Parahippocampal Gyrus	L	31	8	−24	−35	−14	3.47	3.39	0.967	0.037
	Superior Temporal Gyrus	L	63	1	−29	6	−50	3.31	3.25	0.993	0.044
	Fusiform Gyrus	R	7	7	47	−56	−15	3.21	3.15	0.998	0.049
SZ > HC	–	–	–	–	–	–	–	–	–	–	–

**Table 5:** Evidence of cerebrospinal fluid alteration detected by voxel-based morphometry.

Contrast	Regions	L/R	Voxels Size	Clusters Number	MNI coordinates(mm)			Voxel level			
					*X*	*Y*	*Z*	*T*-value	*Z*-value	*p*_FWE corrected_	*p*_FDR corrected_
SZ < HC	–	–	–	–	–	–	–	–	–	–	–
SZ > HC	Third ventricle	L	2,399	6	−2.70	−9.00	2.69	3.43	3.31	0.185	0.012
	Lateral Ventricle	L	2,759	4	−1.80	−4.49	3.00	3.32	3.16	0.377	0.025

## 4. Discussion

The present study aimed to investigate differences in brain volume between patients with schizophrenia and healthy controls using voxel-by-voxel analysis with family-wise error correction method. Our findings indicate significant reductions in gray matter volume in various regions of the brain, including the right inferior frontal gyrus, right middle frontal gyrus, right superior frontal gyrus, right subcallosal gyrus, left middle temporal gyrus, left postcentral gyrus, and left cingulate gyrus. Additionally, we found significant reductions in white matter volume in several brain regions, including the left parahippocampal gyrus, left superior temporal gyrus, right fusiform gyrus, left middle temporal gyrus, left inferior frontal gyrus, right inferior frontal gyrus, right sub-gyral, and right middle frontal gyrus. Moreover, our analysis revealed significant increases in cerebrospinal fluid volume in the left third ventricle and left lateral ventricle regions of schizophrenic patients compared to healthy controls.

### 4.1. Gray and white matter reduction in frontal lobe

The frontal lobe is a critical brain region that is involved in a wide range of cognitive and executive functions, such as decision-making, working memory, attention, planning, and social cognition. Dysfunction in these cognitive domains is a hallmark of schizophrenia and has been linked to abnormalities in the frontal lobe. There was significant reduction of gray and white matter in the inferior frontal gyrus (IFG) and middle frontal gyrus (MFG), as identified in our findings. The IFG is known to be involved in the processing of language, speech production, and semantic processing (53). The MFG, on the other hand, is involved in higher-order cognitive functions, including working memory, attention, and cognitive flexibility (54).

Our findings that identified gray matter (GM) and white matter (WM) reduction in the inferior frontal gyrus (IFG) and middle frontal gyrus (MFG) regions of the frontal lobe provide compelling evidence of the neural basis of this disorder (55). Previous studies have also demonstrated reduced GM volume in the prefrontal cortex (PFC) of schizophrenic patients, including the IFG and MFG. The PFC is known to be critical for executive functions, and its dysfunction has been linked to the cognitive deficits observed in schizophrenia (56). Furthermore, these findings are consistent with other neuroimaging studies that have reported reduced WM integrity in the frontal lobe of schizophrenic patients. A diffusion tensor imaging study found reduced fractional anisotropy, a measure of WM integrity, in the IFG and MFG regions in patients with schizophrenia, further supporting the current VBM findings (57).

The superior frontal gyrus (SFG) is known to play a critical role in cognitive processes, including working memory, attention, and decision-making (58). The subcallosal gyrus (SCG), on the other hand, is involved in emotional regulation and reward processing (59). The current VBM findings support previous research demonstrating altered connectivity in the frontal lobe in schizophrenia. Diffusion tensor imaging studies have identified abnormal white matter tracts connecting the SFG and SCG to other regions of the brain in patients with schizophrenia (60). One study using voxel-based morphometry (VBM) found that schizophrenia patients had significant gray matter reductions in the subcallosal gyrus and superior frontal gyrus compared to controls (61). Similarly, another VBM study reported significant gray matter reductions in the same regions in schizophrenia patients (62). Additionally, a meta-analysis of 42 VBM studies found consistent gray matter reductions in the subcallosal gyrus and superior frontal gyrus of schizophrenia patients (63). These findings suggest that the observed GM reduction in the SFG and SCG may be related to disrupted neural connectivity in these brain regions.

The sub-gyral region of the frontal lobe contains fibers that connect different brain regions and plays a critical role in information processing and integration. The WM volume reduction in this region observed in patients with schizophrenia may reflect a loss of these critical connections and may contribute to the cognitive deficits commonly observed in this disorder. The findings from this study are consistent with previous research that has identified reduced WM volume in other brain regions in patients with schizophrenia. For example, a meta-analysis of VBM studies in patients with schizophrenia found that WM volume reductions were most consistently observed in the frontal and temporal lobes (63). The WM volume reductions observed in the sub-gyral region of the frontal lobe may also be related to abnormalities in other brain regions that are functionally connected to this region. For example, previous research has suggested that disruptions in the prefrontal cortex and its connections with the thalamus and striatum may contribute to the cognitive and emotional symptoms of schizophrenia (64).

### 4.2. Gray and white matter reduction in the temporal lobe

The temporal lobe is a region of the brain that is located on the lateral (side) surface of the cerebral cortex, beneath the temporal bone of the skull. It plays a critical role in processing sensory information such as sound, as well as in memory, emotion, and language comprehension.

The middle temporal gyrus (MTG) plays a critical role in language processing and semantic memory retrieval (65). The observed GM and WM reduction in this region may contribute to the language and memory deficits that are commonly observed in patients with schizophrenia. Previous research has also identified MTG abnormalities in patients with schizophrenia. Structural magnetic resonance imaging (MRI) studies have shown reduced GM volume in the MTG in patients with schizophrenia (66). The current VBM findings of GM and WM reductions in the MTG support previous research demonstrating altered connectivity in this region in schizophrenia. DTI studies have identified abnormal white matter tracts connecting the MTG to other regions of the brain in patients with schizophrenia (67). These findings suggest that the observed GM and WM reduction in the MTG may be related to disrupted neural connectivity in this brain region.

The STG plays a crucial role in processing speech and language, and deficits in this region have been associated with auditory hallucinations in patients with schizophrenia (68). The fusiform gyrus, on the other hand, is involved in facial recognition. Several VBM studies have reported that patients with schizophrenia exhibit decreased white matter (WM) in the superior temporal gyrus (STG) and the fusiform gyrus of the temporal lobe (69). In another study, gray matter degrease was reported in the STG with no white matter changes (70). Furthermore, these findings suggest that the abnormalities in these brain regions may contribute to the pathogenesis of schizophrenia, particularly in the areas of auditory processing and language comprehension, which are known to be impaired in patients with schizophrenia (69, 71).

### 4.3. Gray and white matter reduction in the limbic lobe

The limbic lobe is a group of brain structures that are located in the center of the brain, beneath the cerebrum. It is involved in a variety of functions related to emotion, memory, and motivation. The limbic lobe includes several important structures, including the hippocampus, amygdala, thalamus, hypothalamus, and cingulate gyrus.

One recent VBM study of schizophrenic patients found a significant GM reduction in the cingulate gyrus, a region implicated in emotion processing and cognitive control (72). Our finding is consistent with previous studies that have shown similar GM reductions in the cingulate gyrus of schizophrenic patients (61). The cingulate gyrus is a crucial hub in the default mode network, which is involved in self-referential processing and introspection. The dysfunction of this network has been associated with the symptoms of schizophrenia, such as auditory hallucinations and delusions (73).

Another study reported a significant WM reduction in the parahippocampal gyrus region of the limbic lobe. This region plays a critical role in memory encoding and retrieval and is known to be involved in the pathophysiology of several psychiatric disorders, including schizophrenia (74, 75). A previous study using diffusion tensor imaging (DTI) also reported a reduction of fractional anisotropy (FA), an index of WM integrity, in the parahippocampal gyrus of patients with schizophrenia (76).

### 4.4. Gray matter reduction in postcentral gyrus of the parietal lobe

The postcentral gyrus is located in the parietal lobe. The parietal lobe is a region of the brain located in the upper back part of the cerebral cortex, behind the frontal lobe and above the temporal lobe. It plays a vital role in processing sensory information from the body and in controlling spatial perception and attention. The postcentral gyrus is involved in higher-order sensory processing, including the integration of sensory information from different modalities. Gray matter reductions in this region may therefore have implications for higher-order sensory processing and multisensory integration in schizophrenia.

For example, a study by (77) found that patients with bipolar disorder showed reduced functional connectivity between the postcentral gyrus and other brain regions involved in sensory processing and multisensory integration. In contrast, a VBM study by (78) noted significant increased GM density in the postcentral gyrus, the right thalamus, and the left putamen. Several studies have investigated gray matter reductions in the postcentral gyrus of individuals with schizophrenia. (72) found reduced gray matter volume in the postcentral gyrus of patients with schizophrenia compared to healthy controls. Similarly, (79) reported significant gray matter reductions in the left postcentral gyrus in a VBM analysis report.

These findings suggest that the reduction in gray matter volume in the postcentral gyrus is a consistent feature of schizophrenia. Moreover, this reduction may contribute to the sensory abnormalities experienced by individuals with the disorder. Many studies have found that individuals with schizophrenia have deficits in somatosensory processing, including decreased tactile sensitivity (80) and impaired proprioception (81). These deficits may be related to the gray matter reductions observed in the postcentral gyrus.

### 4.5. Cerebrospinal fluid increase in the third and lateral ventricles of the sublobar

The third ventricle is a narrow cavity located in the midline of the brain, between the two halves of the thalamus, and is part of the ventricular system that produces and circulates CSF throughout the brain. The lateral ventricles are larger cavities located in each hemisphere of the brain and are also part of the ventricular system. The enlargement of these ventricles and the increase in CSF volume suggest disrupted brain fluid dynamics and loss of brain tissue, which is consistent with previous studies (82, 83).

Furthermore, the sublobar region of the brain includes several structures such as the thalamus, basal ganglia, and hypothalamus, which are involved in various cognitive and emotional processes. Dysfunction in these regions has been associated with the symptoms of schizophrenia, such as cognitive deficits and emotional dysregulation (84). Therefore, the CSF volume increases observed in this study may reflect the disruption of these structures and their functional networks in patients with schizophrenia. The MRI findings review of schizophrenic patients in (85) reported a significant difference in CSF volume in the third ventricle and lateral ventricle of the sublobar region. This finding is consistent with other studies that have shown similar CSF volume increases in these regions of the brain in patients with schizophrenia (83, 86).

## 5. Limitations and future directions

One major limitation of the present VBM study is that the cross-sectional design of the study precludes the establishment of a causal relationship between the observed structural brain abnormalities and schizophrenia. Longitudinal studies are needed to determine whether these changes are a result of the illness or represent a pre-existing vulnerability. It is possible that some of the structural brain abnormalities observed in individuals with schizophrenia are a consequence of the illness, while others may represent a pre-existing vulnerability to the disorder. This could be accomplished by using repeated MRI scans and comparing changes in brain structure over time between individuals with schizophrenia and healthy controls.

Furthermore, another drawback to this research is the inability to directly associate clinical symptoms of schizophrenia with the VBM indices during data analysis. Although the study examined structural brain abnormalities in individuals with schizophrenia, it did not investigate how these changes relate to specific clinical symptoms or cognitive deficits. This is important because schizophrenia is a complex disorder with a wide range of symptoms, and it is possible that the observed structural brain abnormalities may be associated with specific symptom clusters or cognitive impairments. This could be accomplished by using advanced statistical techniques, such as machine learning, to identify patterns of brain structure that are associated with specific symptom clusters or cognitive impairments.

In addition, the sample size of the study was relatively small, which limits the generalizability of the findings to other subtypes of the disorder or to individuals with less severe illness. Larger sample sizes are needed to confirm the generalizability of the findings and to explore potential differences in brain structure across subtypes of schizophrenia. Collaborative efforts and data sharing between research groups could help to address this limitation and increase the statistical power of future studies.

Finally, the study used only structural MRI data, which does not provide information on other potential factors such as brain metabolism, connectivity, or functional activity. Future studies should consider using a multimodal approach to investigate the relationship between brain structure, function, and clinical symptoms in individuals with schizophrenia.

## 6. Conclusion

The present VBM study provides further evidence of structural brain abnormalities in individuals with schizophrenia. The theoretical implications of the research findings suggest that structural brain abnormalities in individuals with schizophrenia may contribute to the cognitive and affective deficits associated with the disorder. The identified reductions in gray and white matter volume in multiple regions of the brain, including the frontal, temporal, parietal, and limbic lobes, provide further support for the neurodevelopmental hypothesis of schizophrenia, which suggests that abnormalities in brain development and maturation may underlie the disorder.

The increased cerebrospinal fluid volume in the sublobar region may also provide insight into the underlying neural dysfunction in schizophrenia. Previous research has linked cerebrospinal fluid abnormalities to disrupted neuronal connectivity and impaired neural processing, further supporting the idea that schizophrenia involves a disruption in the neural networks that underlie cognitive and affective processing.

Translational applications of these research findings could include the development of more targeted and effective treatments for individuals with schizophrenia. By identifying specific structural abnormalities in the brain associated with the disorder, clinicians may be able to more accurately diagnose and treat the condition. Additionally, understanding the relationship between these structural abnormalities and clinical symptoms and treatment response may allow for more personalized treatment approaches and improved outcomes for individuals with schizophrenia.

Finally, the present study provides important insights into the structural brain abnormalities associated with schizophrenia and highlights the need for further research to understand the functional consequences of these abnormalities and their potential implications for diagnosis and treatment of the disorder.

## Data availability statement

The original contributions presented in the study are included in the article/supplementary material, further inquiries can be directed to the corresponding author.

## Ethics statement

Ethical review and approval was not required for the study on human participants in accordance with the local legislation and institutional requirements. The patients/participants provided their written informed consent to participate in this study.

## Author contributions

MJA, LQ, CON, AY, HBK, AHJ, and SS substantial contributions to the conception or design of the work. MJA, CON, AY, HBK, AHJ, and SS contributions to the acquisition, analysis or interpretation of data. MJA, CON, AY, HBK, AHJ, and SS drafting the work or revising it critically for important intellectual content. MJA and LQ final approval of the version submitted. All authors contributed to the article and approved the submitted version.

## Funding

This research was supported by the National Natural Science Foundation of China under Grant no. 61471263, 61872267 and U21B2024; the Natural Science Foundation of Tianjin, China, under Grant 16JCZDJC31100, and the Tianjin University Innovation Foundation under Grant 2021XZC-0024.

## Conflict of interest

The authors declare that the research was conducted in the absence of any commercial or financial relationships that could be construed as a potential conflict of interest.

## Publisher’s note

All claims expressed in this article are solely those of the authors and do not necessarily represent those of their affiliated organizations, or those of the publisher, the editors and the reviewers. Any product that may be evaluated in this article, or claim that may be made by its manufacturer, is not guaranteed or endorsed by the publisher.
